# Enhancement of radiation effects by Plantago major in MCF-7 human breast cancer: an animal study

**DOI:** 10.1007/s12672-025-02436-z

**Published:** 2025-05-14

**Authors:** Hongliang Zhao, Hongxiang Gao, Jing Kang, Jiexin Chen, Huiling Ouyang, Peiyao Chen, Hussain Shaik Althaf, Turki Mayudh Alrubie, Maddu Narendra, Kai Wang

**Affiliations:** 1 Department of Hematology & Oncology, Honghui Hospital, Xi An Jiao Tong University, Xian, 710054 People’s Republic of China; 2 Department of Zoology, College of Science, King Saud University, 11451 Riyadh, Saudi Arabia; 3 Department of Biochemistry, Sri Krishnadevaraya University, Anantapur, Andhra Pradesh India

**Keywords:** Radiation, Radiosensitivity, Plantago major, Breast cancer

## Abstract

**Purpose:**

We aimed to evaluate the potential effects of Plantago major (PM) to enhance the radiosensitivity of human breast adenocarcinoma cell line (MCF-7) in an animal model.

**Materials and methods:**

Seventy-two female Balb/c mice were divided into nine groups (8 mice per group) as follows: MCF-7 breast cancer control group, MCF-7+low dose of PM (1000 mg/kg), MCF-7+high dose of PM (1500 mg/kg), MCF-7+3 Gy superficial X-ray, MCF-7+5 Gy superficial X-ray, MCF-7+1000&1500 mg/kg of PM with 3 and 5 Gy irradiations. For each treatment group, micronuclei in 500 binucleate MCF-7 cells from a minimum of three experiments were counted. The alkaline comet assay was used to calculate % DNA in the tail and % of apoptotic comets. The tumor size in the treated groups (3 mice per group) was assessed at 4- and 8-weeks post-treatment.

**Results:**

The number of total micronuclei and binucleated micronuclei in the PM and/or irradiation treated groups was significantly higher than in the control group (P < 0.05). Irradiation+PM resulted in a significant increase treatment effect compared to the irradiation-only groups (P < 0.01). In addition, the higher PM concentration had a significantly higher number of micronucleated binucleate cells when combined with 5 Gy irradiation (P = 0.022). Irradiation alone or in combination with PM resulted in significant increases in percentages of DNA in tail and apoptotic comet values compared to the PM-only treatment groups (P < 0.02). Combining 5 Gy of irradiation with 1000 mg/kg of PM led to a 26% reduction in tumor size (0.28±0.04 vs. 0.38 ± 0.03) after 4 weeks, and combining 5 Gy of irradiation with 1500 mg/kg of PM resulted in a 40% decrease in tumor volume after 4 weeks (0.21±0.04 vs. 0.35±0.03).

**Conclusion:**

PM extract at both doses demonstrated an antitumor effect on induced MCF-7 breast cancer tumors, with this effect being enhanced when combined with irradiation.

## Introduction

Cancer is the primary cause of death in developed nations and the second most common cause of death in developing nations [1]. Among various types of cancer, breast cancer is particularly common and is the most prevalent cancer among women worldwide [2, 3]. The primary treatment methods for breast cancer include radiotherapy, hormone therapy, chemotherapy, and surgery [4]. While radiotherapy is effective, it can also cause side effects, such as the induction of secondary cancers and radiation injuries to sensitive organs due to the use of ionizing radiation [5, 6].

Various irradiation therapy strategies are used to deliver the prescribed dose to the tumor (or target tissue) while minimizing exposure to radiosensitive organs and normal tissues. Advances in imaging and modern radiotherapy techniques are examples of non-pharmacological methods that have improved treatment accuracy [7, 8]. Pharmaceutical strategies involve the use of radioprotectors and radiosensitizers to mitigate free radical damage during radiotherapy. These agents can potentially reduce normal tissue toxicity, decrease radiotherapy side effects and complications, and lower the probability of inducing secondary cancers [9].

Plantago major (PM) is a medicinal herb historically used for various treatments [10, 11], such as viral illnesses, including colds, influenza, and viral hepatitis [12]. PM’s primary therapeutic benefits are attributed to its antioxidant activity, which helps prevent cell damage and cancer formation [13]. Furthermore, its anticancer properties have been reported in several studies [10–15]. Methanolic extracts of PM have shown anticancer effects in several human cancer cell lines, including renal adenocarcinoma (TK-10), breast adenocarcinoma (MCF-7), and melanoma (UACC-62) [14–16]. PM contains significant amounts of flavones, flavonoids, and luteolin, which are the main contributors to its anticancer properties [17–19]. Additionally, PM extract has demonstrated cytotoxic effects on cervical (SQC-UISO) and ovarian (OVCAR) cancer cells, reducing UISO cells by 59% and OVCAR cells by 82% [15].

The MCF-7 cell line is a human breast cancer model that expresses estrogen, progesterone, and glucocorticoid receptors. These cells are widely used in both in vivo and in vitro studies due to their ideal characteristics specific to the mammary epithelium [20]. Our search indicates that no in vivo studies have clarified the positive effects of PM in combination with radiotherapy on MCF-7. Therefore, we aimed to evaluate the potential of PM to enhance the radiosensitivity of MCF-7 cancer cells in Balb/c mice.

## Materials and methods

### PM extraction

The dried PM leaves, sourced from the National Institute of Pharmaceutical and Biological Products, were rinsed with tap water and allowed to dry at room temperature. One hundred grams of the pulverized leaves were then extracted using a Soxhlet apparatus for 48 h with 1 L of methanol. The insoluble material was removed by filtration with Whatman No. 3 paper and subsequently dried with a rotary evaporator (Eyela N-1000, Canada). The extract was collected and stored in a refrigerator at 4 °C until further use. Finally, the extraction yield was approximately 42%.

### MCF-7 cell line

The MCF-7 cell line (ATCC HTB-22), an epithelial-origin human breast cancer cell line, was acquired from the Chinese Academy of Medical Sciences Cancer Institute (Beijing, China). The cells were cultured in Minimal Essential Medium (MEM) with Earle’s salts (GibcoBRL, Life Technologies, Rockville, MD), supplemented with 10% fetal bovine serum (FBS, BioWhittaker, Walkersville, MD) and antibiotics (penicillin, gentamycin, and fungizone).

### Animals

Seventy-two female Balb/c mice, aged 6–8 weeks and weighing an average of 32±4 g, were obtained from the Chinese Academy of Medical Sciences Laboratory Animal Center. They were kept in standard cages with a 12-h light–dark cycle for at least 1 week prior to the start of the study, with unrestricted access to food and water. All animal experiments were approved by the national Animal Care and Use Committee. The mice were divided into nine groups (5 mice per group for micronucleus and alkaline comet assays+3 additional mice for tumor size evaluation) as follows:

1. MCF-7 breast cancer control group: The animals with the MCF-7 breast cancer model without treatment.
2. MCF-7 breast cancer with low dose of PM (1000 mg/kg) group: Mice with the breast cancer model, treated with a daily oral dose of 1000 mg/kg PM for 6 consecutive days.
3. MCF-7 breast cancer with high dose of PM (1500 mg/kg) group: The animals with the MCF-7 breast cancer model, treated with a daily oral dose of 1500 mg/kg PM for 6 consecutive days.
4. MCF-7 breast cancer with 3 Gy irradiation: The animals with the breast cancer model, treated with one fraction of 3 Gy superficial X-rays.
5. MCF-7 breast cancer with 5 Gy irradiation: The animals with the breast cancer model, treated with one fraction of 5 Gy superficial X-rays.
6. MCF-7 breast cancer with 1000 mg/kg of PM and 3 Gy irradiation: The animals with the breast cancer model, treated with a daily oral dose of 1000 mg/kg PM for 6 consecutive days, followed by 3 Gy of superficial X-rays.
7. MCF-7 breast cancer with 1500 mg/kg of PM and 3 Gy irradiation: The animals with the breast cancer model, treated with a daily oral dose of 1500 mg/kg PM for 6 consecutive days, followed by 3 Gy of superficial X-rays.
8. MCF-7 breast cancer with 1000 mg/kg of PM and 5 Gy irradiation: Mice with the breast cancer model, treated with a daily oral dose of 1000 mg/kg PM for 6 consecutive days, followed by 5 Gy of superficial X-rays.
9. MCF-7 breast cancer with 1500 mg/kg of PM and 5 Gy irradiation: Mice with the breast cancer model, treated with a daily oral dose of 1500 mg/kg PM for 6 consecutive days, followed by 5 Gy of superficial X-rays.

To establish the mouse model, the cells were diluted with phosphate-buffered saline (PBS) to a concentration of 5×10^7^ cells/ml. Subsequently, 5×10^6^ MCF-7 human breast cancer cells in 100 μl of PBS were injected subcutaneously into the left back armpit of each mouse, based on preliminary experimental results [21]. One week after inoculation, the implanted tumors developed into hard nodular masses measuring 0.3–0.4 cm in diameter, at which point the experimental treatments were initiated.

### Irradiation

Irradiation was performed one hour after the final dose of PM administration in the relevant groups. The mice were exposed to 3 and 5 Gy of radiation using a superficial X-ray machine (Phillips), operated at 250 kVp and 15 mA with a 1 mm copper filter. Exposure doses were measured with an ionization chamber to determine the radiation time. The absorbed doses were measured in air using appropriate temperature and pressure correction factors, along with Roentgen to Gy conversion factors. The superficial X-ray beams were precisely conformed to irradiate only the tumor beneath the skin, with about 5 mm margins, sparing other normal skin areas.

After 24 h of irradiation, the mice were anesthetized with 100 mg/kg of ketamine and 10 mg/kg of xylazine. They were then sacrificed by cervical dislocation, and the breast cancer tumors were removed for the micronuclei test. Additionally, 3 extra mice from each of the irradiated and treated groups were kept for 8 weeks post-irradiation, and the tumor sizes were measured at 0, 4, and 8 weeks after irradiation.

### Micronucleus assay

This test was conducted on MCF-7 tumor cells using the method described by Mishra et al. [22]. To obtain single-cell suspensions, tumor tissues were excised, minced into small fragments, and enzymatically digested with a solution of collagenase (0.1% w/v) and DNase I (0.01% w/v) at 37 °C for 30 min under continuous agitation. The resulting cell suspensions were then filtered through a 70-μm cell strainer to remove debris. After isolation, the MCF-7 cells were washed with PBS and maintained under appropriate culture conditions until further processing.

In summary, the harvested cells were suspended and placed in a centrifuge tube along with FBS. The cells were then collected by centrifugation at 1000 rpm for 10 min at 4 degrees Celsius. The resulting smears were transferred onto slides and left at room temperature. After 24 h of air-drying, the smears were stained with May-Grunwald/Giemsa stain. For each treatment group, micronuclei in 500 binucleate MCF-7 cells from a minimum of three experiments were counted. The mitotic rate was determined as the percentage of binucleate cells (mean±standard deviation, n = 3).

### Alkaline comet assay

The DNA damage in tumor tissue was assessed using the alkaline comet assay method outlined by Kumar et al. [23]. As the tumor was located subcutaneously, tumor-derived cells were isolated from each mouse at the selected time points of 0, 2, and 24 h after treatment. To obtain single-cell suspensions, tumor tissues were excised, minced into small fragments, and enzymatically digested with a solution of collagenase (0.1% w/v) and DNase I (0.01% w/v) at 37 °C for 30 min under continuous agitation. The resulting cell suspensions were then filtered through a 70-μm cell strainer to remove debris. After isolation, the MCF-7 cells were washed with PBS and collected by trypsinization.

In summary, cells were suspended in 0.7% low-melting-point agarose and layered onto frosted slides precoated with 1% normal-melting-point agarose. The slides were then placed in a lysis solution (1% N-sodium lauryl sarcosinate, 2.5 M NaCl, 10 mM Tris, 30 mM Na2EDTA, 1% Triton X-100, and 10% DMSO) for 1 h at 48 °C. Subsequently, the slides were immersed in an alkaline buffer (200 mM NaOH, 100 mM Na2EDTA, pH 13.0) for 20 min, followed by alkaline electrophoresis (300 mM NaOH, 1 mM Na2EDTA, pH 13.0) at 0.7 V/cm, 300 mA for 20 min at 48 °C. After electrophoresis, the slides were washed with 0.4 M Tris base buffer (pH 7.4), fixed in 70% ethanol for 15 min, dehydrated on a hot plate, and stored in a dry place at room temperature. Before analysis, the slides were rehydrated in distilled water, stained with propidium iodide (25 mg/mL) for 10 min, and examined at 203 magnifications using an automated scanning fluorescence microscope Metafer 4 (Meta-system, Germany) to assess the migration of damaged DNA. The MetaCyte Comet Scan module was used for automated comet analysis, scanning at least 1000 nuclei to calculate the relevant parameters, including %DNA in the tail to measure migrated nuclear DNA of cells. Another crucial parameter, % of apoptotic comets, was determined by comets with more than 60% DNA in the tail.

### Tumor size measurement

The tumor size in the treated groups (with 3 mice in each group) was assessed at 4- and 8-weeks post-treatment. Measurements of tumor length and width were taken using calipers. The preclinical tumor volume is commonly calculated using the formula

$$ V\, = \,0.5\, \times \,L\, \times \,W^{ \wedge } 2 $$

where V represents tumor volume, L represents tumor length, and W represents tumor width. This method is straightforward, quick, and reasonably accurate [24].

### Statistical analysis

The data analysis was conducted using SPSS 22 (SPSS Inc., Chicago, Illinois, USA) software. Variations among the groups for each specified parameter were evaluated using a one-way ANOVA statistical test, followed by Tukey’s multiple comparison post hoc test. A significance level of 95% confidence was adopted, and P-values less than 0.05 were considered indicative of significant differences.

## Results

### Micronucleus assay

**Fig. 1:**
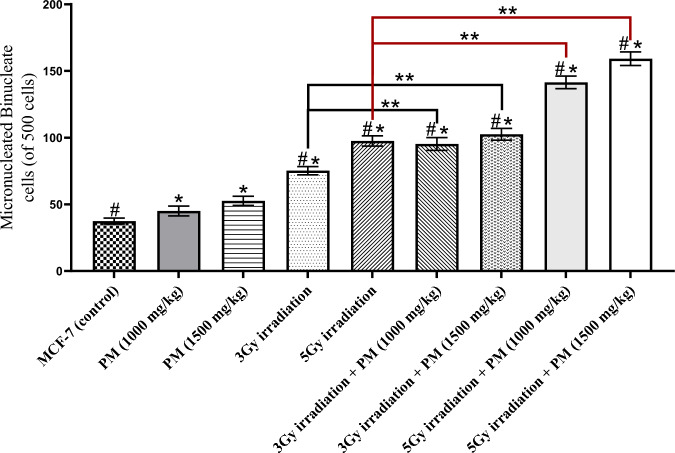
Mean+SD of micronucleated bi-nucleate cell number (in 500 cells) among the experimental groups. The significant differences with the control group were determined with “*” sign. The significant differences with the PM only treated groups were demonstrated with “#” sign. The “**” sign illustrates the significant differences between the groups determined by the bracket

**Fig. 2:**
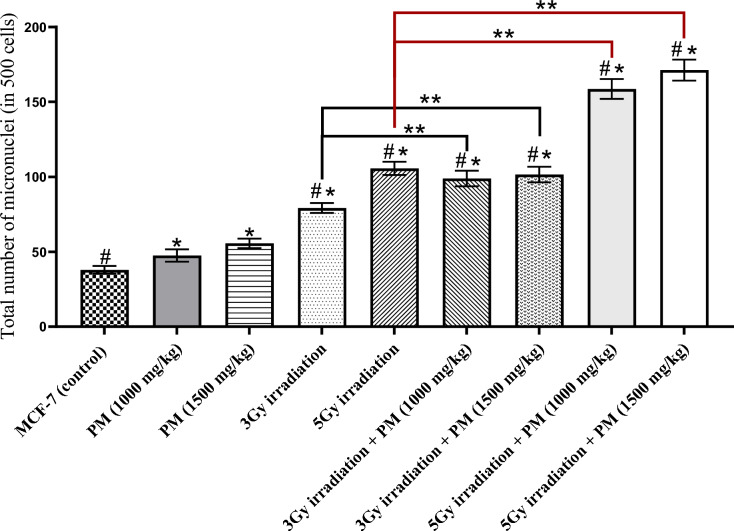
Mean+SD of total number of micronuclei (in 500 cells) among the experimental groups. The significant differences with the control group were determined with “*” sign. The significant differences with the PM only treated groups were demonstrated with “#” sign. The “**” sign illustrates the significant differences between the groups determined by the bracket

The number of micronucleated binucleate cells (in 500 cells) is presented in Fig. 1. The results showed that PM treatment (at both evaluated concentrations) significantly increased binucleate micronucleus-forming activity. Figure 2 also illustrates the total number of micronuclei (in 500 cells) among the evaluated groups (P < 0.05). The number of total micronuclei in the PM-treated groups was significantly higher than in the control breast cancer group (P < 0.02). Treatment with a higher concentration of PM resulted in a higher number of total micronuclei and binucleated micronuclei, but these differences were not statistically significant (P > 0.05).

Irradiation with 3 and 5 Gy of X-rays resulted in significantly higher micronucleus formation compared to the control and PM-only treatment groups (Figs. 1 and 2) (P < 0.001). Combining irradiation with PM also resulted in a significant increase compared to the irradiation-only groups (P < 0.01). Although the higher concentration of PM (1500 mg/kg) combined with irradiation (3 Gy or 5 Gy) did not show a significant difference from the lower concentration (1000 mg/kg) in the total number of micronuclei (P > 0.05), the higher concentration had a significantly higher number of micronucleated binucleate cells when combined with 5 Gy irradiation (P = 0.022).

**Fig. 3:**
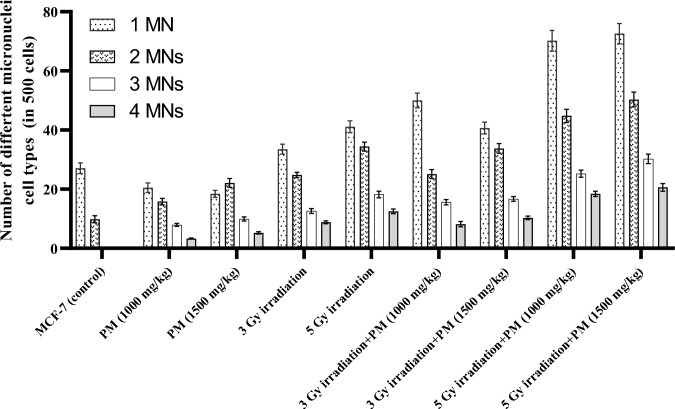
Mean+SD values of the number of different micronuclei cell types (in 500 cells) among the experimental groups

Figure 3 shows the distribution of micronuclei types based on the number of micronuclei observed in a cell among the evaluated groups. The results indicated that the control breast cancer group did not have any cells with three or more micronuclei. In contrast, treatment with PM (at both concentrations), irradiation (at both doses), and the combination of PM with irradiation resulted in the production of cells with three, four, or more micronuclei.

The number of cells with three and four (or more) micronuclei was significantly higher in the high-concentration PM-only treatment group compared to the lower concentration PM-only treatment group (P = 0.011). The number of cells in all categories (1–4 micronuclei) was also significantly higher in the 5 Gy irradiated groups compared to the 3 Gy irradiated groups (P < 0.05). The combination of irradiation with PM administration resulted in a significant increase in the number of cells in all categories (1–4 micronuclei) compared to the irradiation-only groups (P < 0.02), except for the number of cells with two micronuclei in the 3 Gy irradiation group (P = 0.108).

### Alkaline comet assay

**Fig. 4:**
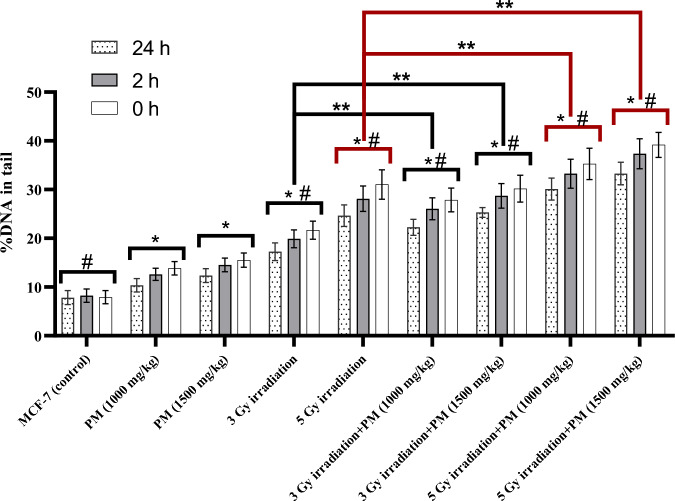
Mean+SD values of DNA in tail percentage among the experimental groups at three evaluated time points including 0, 2, and 24 h after treatment. The significant differences with the control group were determined with “*” sign. The significant differences with the PM only treated groups were demonstrated with “#” sign. The “**” sign illustrates the significant differences between the groups determined by the bracket

**Fig. 5:**
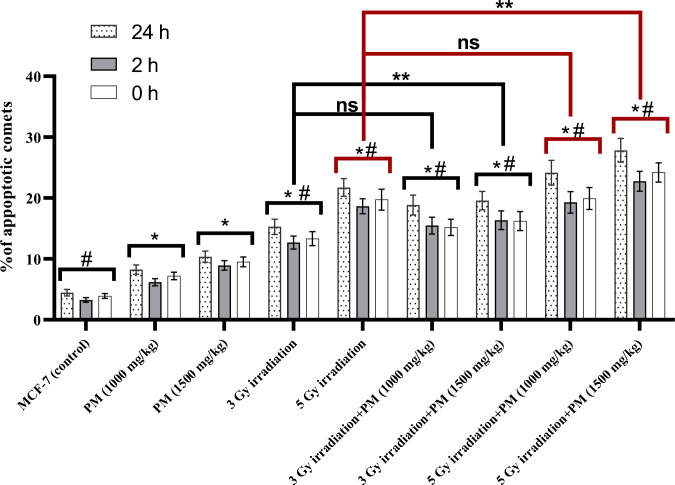
Mean+SD values of apoptotic comet percentage among the experimental groups at three evaluated time points including 0, 2, and 24 h after treatment. The significant differences with the control group were determined with “*” sign. The significant differences with the PM only treated groups were demonstrated with “#” sign. The “**” sign illustrates the significant differences and “ns” shows the non-significant differences between the groups determined by the bracket

Percentages of DNA in tail and apoptotic comets among the evaluated groups are presented in Figs. 4 and 5, respectively, at three time points: immediately, 2 h, and 24 h after treatment. The results of statistical analysis showed that both concentrations of PM had significantly higher percentages of DNA in tail and apoptotic comet values compared to the control group (P < 0.05). Furthermore, irradiation alone or in combination with PM resulted in significant increases in these parameters compared to the PM-only treatment groups (P < 0.02).

When excluding the control group, the percentages of DNA in tail values decreased after 2 and 24 h of treatment, although this decrease was not significant after 2 h (P > 0.07). However, the percentages of DNA in tail values significantly decreased after 24 h for all groups (P < 0.05), except for the combination of 5 Gy irradiation with 1000 mg/kg of PM group (P = 0.0606) (Fig. 4).

The percentage of apoptotic comets showed a non-significant decrease after 2 h (P > 0.10) and a significant increase after 24 h in almost all the evaluated groups (P < 0.05), except for the groups treated with 5 Gy irradiation only and PM only (P > 0.085). It is noteworthy that the combination of irradiation and PM administration resulted in significantly higher percentages of DNA in tail and apoptotic comets at all evaluated time points compared to irradiation alone (P < 0.03). Additionally, comparing the combination groups with higher concentrations of PM showed non-significantly higher values of the DNA damage parameters in most of the evaluated time points (P > 0.05).

**Fig. 6:**
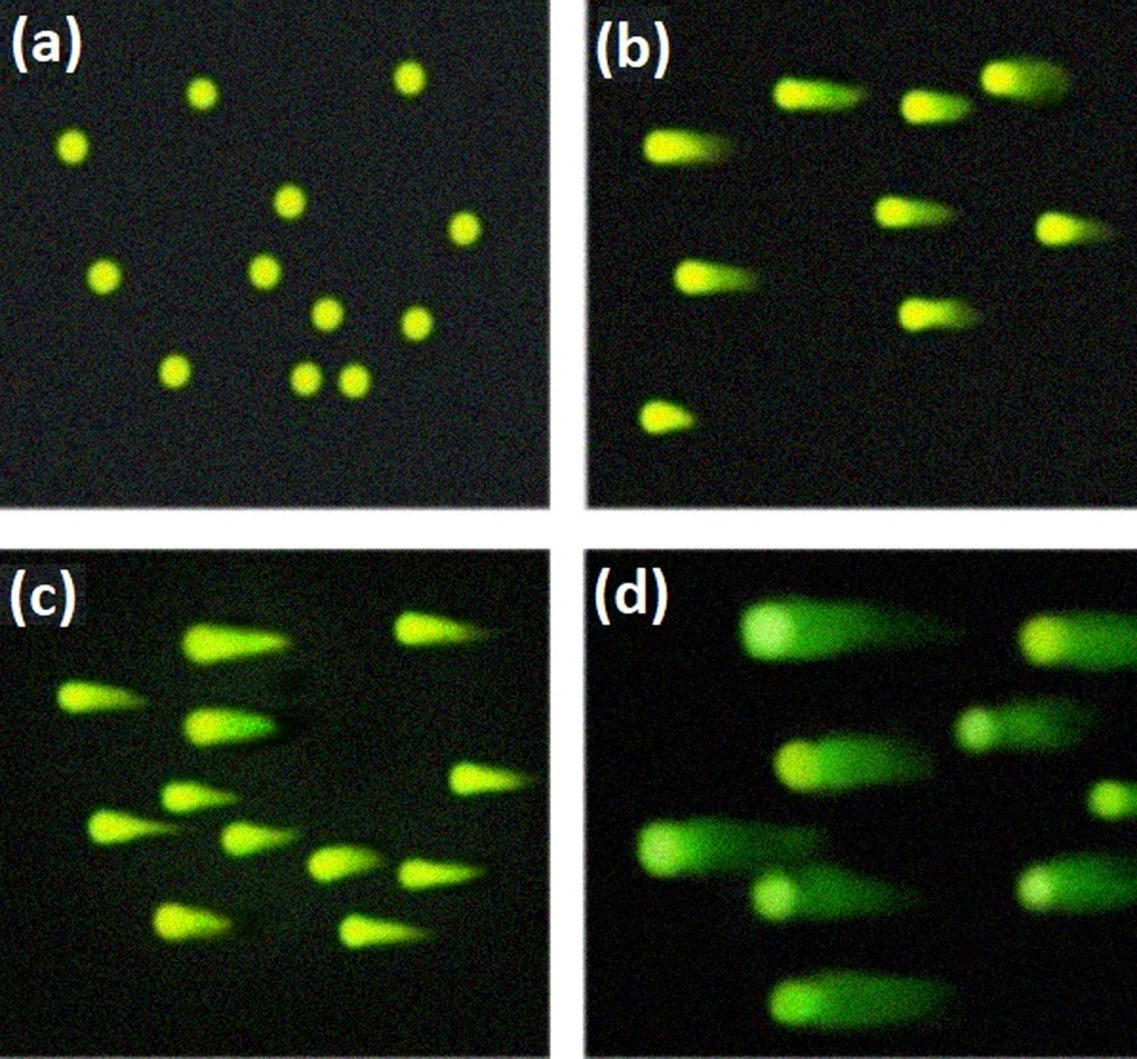
Examples of the alkaline comet assay. **a** MCF-7 breast cancer control group, **b** MCF-7+high-dose PM (1500 mg/kg), **c** MCF-7+5 Gy superficial X-ray, and **d** MCF-7+1500 mg/kg PM with 5 Gy irradiation

Examples of the alkaline comet assay for various groups, including the MCF-7 breast cancer control group, MCF-7+high-dose PM (1500 mg/kg), MCF-7+5 Gy superficial X-ray, and MCF-7+1500 mg/kg PM with 5 Gy irradiation, are shown in Fig. 6.

### Tumor size measurement

The mean and standard deviation values of tumor volumes measured before treatment, and at 4 and 8 weeks after treatment, are presented in Table 1. Statistical analysis showed a significant increase in tumor volume after 4 weeks (142% increase) and 8 weeks (413% increase) for the control group (p < 0.001).

**Table 1:** Mean+SD values of the tumor volumes measured before the treatment, and at 4- and 8-weeks after the treatment

Experimental groups	Tumor volume (cc)		
	Before treatment	After treatment (4 weeks)	After treatment (8 weeks)
MCF-7 breast cancer (control)	0.38±0.03	0.92±0.06	1.95±0.07
PM (1000 mg/kg)	0.33±0.02	0.61±0.05	1.18±0.05
PM (1500 mg/kg)	0.37±0.02	0.59±0.04	0.91±0.06
3 Gy irradiation	0.35±0.03	0.48±0.04	0.78±0.05
5 Gy irradiation	0.36±0.03	0.45±0.05	0.71±0.05
3 Gy irradiation+PM (1000 mg/kg)	0.38±0.02	0.42±0.04	0.65±0.06
3 Gy irradiation+PM (1500 mg/kg)	0.34±0.02	0.39±0.05	0.55±0.05
5 Gy irradiation+PM (1000 mg/kg)	0.38±0.03	0.28±0.04	0.42±0.06
5 Gy irradiation+PM (1500 mg/kg)	0.35±0.03	0.21±0.04	0.37±0.06
P-value	0.405	0.004	0.001

Treatment with PM (at both concentrations), irradiation (at both doses), and the combination of irradiation and PM resulted in significantly lower tumor growth after 4 and 8 weeks (P < 0.001). Specifically, combining 5 Gy of irradiation with 1000 mg/kg of PM led to a 26% reduction in tumor size (0.28±0.04 vs. 0.38±0.03) after 4 weeks, and combining 5 Gy of irradiation with 1500 mg/kg of PM resulted in a 40% decrease in tumor volume after 4 weeks (0.21±0.04 vs. 0.35±0.03).

Tumor control (reduced tumor growth) values at 4 and 8 weeks post-treatment were significantly better in irradiated-only groups compared to groups treated with only PM (P < 0.05). However, the differences were not significant between the PM-treated groups (with low and high concentrations) (P > 0.09). Irradiation combined with PM administration showed better tumor control compared to irradiation alone (with the same dose of X-rays). Although the differences were significant after 8 weeks of treatment (P < 0.05), they were not significant at 4 weeks (P > 0.10).

## Discussion

This study evaluated the PM effects on a human breast cancer model when combined with radiotherapy. This herb has been used for centuries to treat various health conditions, including colds, skin ailments, and infectious diseases [11, 25–28]. Reports suggest that PM can help balance cellular energy by activating cyclic adenosine monophosphate (cAMP) and reduce inflammation by influencing the NF-κB and mitogen-activated protein kinase (MAPK) signaling pathways [29]. PM extract has demonstrated efficacy as a chemoprophylactic and antimetastatic agent against several malignancies, inhibiting the proliferation of various cancer cell lines [16, 30]. The National Cancer Institute (NCI) has recommended PM for its significant activity in inhibiting cell proliferation in breast adenocarcinoma and melanoma cell lines [30].

Our study revealed that administering PM extract at doses of 1000 mg/kg and 1500 mg/kg significantly increased the number of total micronuclei and binucleated micronuclei in a dose-dependent manner compared to the untreated cancer group. Several studies have evaluated the radio-sensitizing effects of natural compounds such as Curcumin [31–33], Resveratrol [34, 35], Withaferin A [36, 37], Celastrol [38], Ursolic Acid [39, 40], Flavopiridol [41, 42], Berberin [43, 44], and Genistein [45, 46] on the treatment of various cancers. However, the combination (radio-sensitizer) effect of PM with radiation had not been previously investigated, making this study the first to evaluate this effect. Additionally, our literature review indicates that the combination of PM with other cancer treatment methods has not been previously explored. Our results also demonstrated that combining PM with irradiation produced greater antitumor effects—measured by micronucleus, alkaline comet, and tumor size assays—compared to using PM or irradiation alone. However, increasing the PM dose from 1000 to 1500 mg/kg did not result in significant changes across all assessed parameters. Consistent with our findings, nearly all previous studies have reported enhanced antitumor effects when combining natural plant compounds with radiotherapy for various cancers. Several studies have explored PM doses ranging from 25 to 2000 mg/kg [11, 47–49]. In this study, we selected relatively high doses to evaluate their therapeutic effects.

The effects of PM on MCF-7 cancer cells and normal human umbilical vein endothelial cells (HUVECs) were evaluated for cytotoxic activity in vitro [18]. It was reported that PM leaf extracts decreased MCF-7 cell proliferation but with minimal effects on normal HUVECs at various doses (50, 100, 250, 500 and 1000 µg/mL) at 24 and 48 h after treatment. The PM plant extract significantly inhibited MCF-7 cell proliferation while having only a slight inhibitory effect on HUVEC cell proliferation. In fact, the inhibitory effect of PM extracts was greater on MCF-7 cells than on HUVEC cells.

Poor et al. [50] examined the effect of PM seed extracts on the survival of MCF-7 cells using the MTT assay. They analyzed the impact of PM concentrations of 1, 1.5, 2, 2.5, 5, 10, and 15 μg/mL on cell growth 24 h after seeding the cells into 96-well microtiter plates. Their findings indicated that PM extract concentrations of 1, 1.5, and 2 μg/mL did not exhibit considerable toxic effects on MCF-7 cancer cells. However, significant toxic effects were observed at doses higher than 5 μg/mL. Ozaslan et al. [51] examined the antitumor potential of PM extract in 30 male Balb/C mice with Ehrlich ascites tumors. PM extract, at concentrations of 1%, 2%, and 3%, was administered orally at 0.1 ml/day/mouse for 10 alternate days in the treatment groups. Their pathological analysis indicated that PM extract, particularly at a concentration of 1%, exerted an inhibitory effect on the tumor. Similar to our findings, they concluded that PM treatment demonstrates an inhibitory effect on tumor cells. In another study, Rahamooz-Haghighi et al. [52] investigated the anticancer effects of ethanolic, methanolic, and acetonic extracts of PM on HEK-293, HCT-116, and SW-480 cell lines. Their results indicated that PM extract contains a diverse range of metabolites, including 2,4-di-tert-butylphenol, gentisic acid, linoleic acid, isoborneol, camphor, methyl ester/linoleic acid ester, methyl stearate, p-cymene, stearophanic acid, and trans-anethole. These compounds could potentially be utilized as precursors for developing antitumor drugs.

It was also reported that PM can ameliorates the side effects of cancer treatment [53, 54]. Soltani et al. [54] demonstrated that PM extract can reduce radiation-induced mucositis in patients with head and neck cancers undergoing radiation therapy. Although there is limited research on the ameliorative effects of PM on radiotherapy damages and side effects, future studies could help establish the safety and efficacy of using PM during or after radiation therapy.

The main biochemical components of PM responsible for its anti-inflammatory and anti-tumor effects are ursolic acid, oleanolic acid, and α-linolenic acid [55]. These compounds have been shown to exert inhibitory effects on COX-2-catalyzed prostaglandin production. Additionally, luteolin-7-O-glucoside, the major flavonoid in this plant, inhibits various human cancer cell lines by acting as a potent DNA topoisomerase I poison [30]. It inhibits cancer cell proliferation and induces apoptosis by inhibiting the RSK1 pathways. Moreover, luteolin found in PM extract has the ability to suppress leukocyte migration, contributing to its anti-inflammatory effects [56].

Some human studies have explored the use of PM in cancer treatment, including research by Soltani et al. [54] and Cabrera-Jaime et al. [53] on its effectiveness in treating mucositis in head and neck cancer patients. Furthermore, previous studies, as well as our own, have reported its potential therapeutic effects in treating various cancers. In this study, we demonstrated the potential synergistic effects of combining this herbal agent with radiotherapy. Therefore, future studies can be designed to further evaluate the clinical potential of our treatment strategy.

Several studies have reported the non-toxic effects of PM and the absence of side effects on normal tissues in animal models [47, 49]. Therefore, our focus was on evaluating the therapeutic effects of PM (with and without irradiation) in mice with MCF-7-induced tumors, and we did not assess potential side effects or damage to normal tissues. Additionally, we aimed to ensure targeted radiation exposure to the tumor while sparing peripheral normal tissues. Based on our method, we believe that normal tissue damage is minimal; however, this aspect can be further evaluated in future studies.

Although the PM doses selected for this study were based on previous similar research, future studies could explore a broader range of irradiation doses and PM concentrations. Additionally, other cancer cell lines could be investigated to assess the antitumor effects of PM, both alone and in combination with radiotherapy.

## Conclusion

The results of the micronucleus and alkaline comet assays, along with tumor size measurements, demonstrate that treatment with PM extract (at doses of 1000 and 1500 mg/kg) or X-ray irradiation (3 and 5 Gy) alone produces an antitumor effect on MCF-7 breast cancer tumors induced in the Balb/c mouse model. Although combining PM with X-ray irradiation enhances the therapeutic effects compared to each treatment alone, increasing the PM dose from 1000 to 1500 mg/kg did not show significant effects across all assessed parameters. Further investigations into the ameliorative effects of PM on normal tissues during or after radiotherapy could pave the way for its clinical applications.

## Data Availability

All data used in this study are available from the corresponding author upon reasonable request.
